# Overexpression of native IF1 downregulates glucose-stimulated insulin secretion by pancreatic INS-1E cells

**DOI:** 10.1038/s41598-020-58411-x

**Published:** 2020-01-31

**Authors:** Anežka Kahancová, Filip Sklenář, Petr Ježek, Andrea Dlasková

**Affiliations:** Department of Mitochondrial Physiology, No. 75, Institute of Physiology, The Czech Academy of Sciences, Prague, Czech Republic

**Keywords:** Energy metabolism, Energy metabolism

## Abstract

We have previously reported that transient knock-down of ATPase inhibitory factor 1 (IF1) by siRNA upregulates ATP levels and subsequently augments insulin secretion in model pancreatic β-cells INS-1E. Here we investigated how long-term IF1-overexpression impacts pancreatic β-cell bioenergetics and insulin secretion. We generated INS-1E cell line stably overexpressing native IF1. We revealed that IF1 overexpression leads to a substantial decrease in ATP levels and reduced glucose-stimulated insulin secretion. A decrease in total cellular ATP content was also reflected in decreased free ATP cytosolic and mitochondrial levels, as monitored with ATeam biosensor. Consistently, cellular respiration of IF1-overexpressing cells was decreased. 3D structured illumination microscopy (SIM) revealed a higher amount of insulin granules with higher volume in IF1-overexpressing cells. Similar effects occurred when cells were incubated at low glucose concentrations. Noteworthy, activation of PKA by dibutyryl cAMP entirely abolished the inhibitory effect of IF1 overexpression on ATP production and insulin secretion. Mitochondrial network morphology and cristae ultrastructure in INS-1E overexpressing IF1 remained mostly unchanged. Finally, we show that INS-1E cells decrease their IF1 protein levels relative to ATP synthase α-subunit in response to increased glucose. In conclusion, IF1 actively downregulates INS-1E cellular metabolism and reduces their ability to secrete insulin.

## Introduction

Adequate secretion of insulin by pancreatic β-cells in response to glucose and other secretagogues is necessary to maintain blood glucose homeostasis. The ATP/ADP ratio is recognized as one of the key factors in promoting insulin secretion by pancreatic β-cells after glucose stimulation^1–6^. Increase in the ATP/ADP ratio leads to a closure of ATP-sensitive potassium channels (K_ATP_) and the subsequent depolarization of the plasma membrane leads to the opening of voltage-gated calcium channels. The increase of intracellular calcium is then the triggering signal for exocytosis of insulin secretory granules. Besides the role in the inhibition of K_ATP_ channel, ATP provides energy for the maturation of insulin granules, their transport along the cytoskeletal network and also regulates protein kinase A (PKA) and AMP-activated protein kinase (AMPK) activity. Both of the latter mentioned signalling pathways are involved in the regulation of insulin secretion^7–12^.

In the majority of cell types, the ATP/ADP ratio is dictated by the energy demand of the cell. On the contrary, pancreatic β-cells are thought to have a specific regulation of metabolic pathways which ensures that the ATP/ADP ratio is governed by the substrate availability^13,14^. When glucose levels are increased in the bloodstream, glucose is transported (by GLUT2 in rodents, by GLUT1−GLUT3 in humans) to the cytosol of pancreatic β-cells and further metabolized by glycolysis, citric acid cycle and oxidative phosphorylation to produce ATP. The conversion of glucose to glucose-6-P by hexokinase-4 (glucokinase) is recognized as the rate-limiting step of these catabolic reactions. Glucokinase is thus thought to be responsible for the specific metabolism of pancreatic β-cells^15–17^.

Nonetheless, it is conceivable that other regulatory check-points must be present in catabolic pathways of pancreatic β-cells, which enable the cell to adjust metabolic flux and ATP production. In this work, we focused on ATPase inhibitory factor IF1 and its impact on the regulation of oxidative phosphorylation (OXPHOS) in pancreatic β-cells. In general, the activity of ATP synthase has to be precisely controlled to maintain the balance between ATP levels and proton motive force Δ*p* (specifically the membrane potential ΔΨ_m_ component), established by the respiratory chain pumps. Both the lack of ATP and a sudden decrease in ΔΨ_m_ could be detrimental^18–20^.

The ATPase inhibitory factor 1, an endogenous regulator of the ATP synthase^21,22^, is consensually accepted as a factor involved in the control of this balance, under specific conditions when the ATP synthase consumes ATP to generate ΔΨ_m_. Such a situation occurs under conditions of severe hypoxia or starvation. IF1 inhibits this reverse mode of ATP synthase to prevent total ATP depletion^23–25^. Several previous studies proposed that IF1 can also inhibit ATP synthesis^26–31^. Noteworthy, the recent work, which resolved a structure of ATP synthase tetramers from the pig (*Sus scrofa domesticus*) by cryo-EM, revealed that the two distinct dimers are joined together by IF1 homodimers in an antiparallel orientation^32^. These ATP synthase tetramers are in a putatively inactive conformation. This discovery stresses the importance of further research to unveil the relation between ATP synthase oligomerization, the functional role of IF1 and the regulation of the ATP synthase activity.

We have previously shown that IF1 knock-down by siRNA leads to an increase in ATP levels and consequently upregulated insulin secretion in model pancreatic β-cells (INS-1E)^33^. Cells with downregulated IF1 levels had disturbed but apparently enhanced sensing of glucose, when even non-stimulatory glucose levels led to substantial ATP production and subsequent insulin secretion. Consistently with our previous work, we now demonstrate that overexpression of native IF1 levels critically downregulates ATP levels and inhibits glucose-stimulated insulin secretion (GSIS) in INS-1E cells. This effect is prevented when cells are incubated with dibutyryl cAMP (dbcAMP), i.e.when protein kinase A (PKA) signalling is activated. Moreover, we also show that IF1 protein levels relative to ATP synthase α-subunit in INS-1E cells are reciprocally dictated by the glucose availability, thus decreasing at increasing glucose.

## Results

### IF1 overexpression impairs INS-1E cells growth and protects against cell death

**Figure 1:**
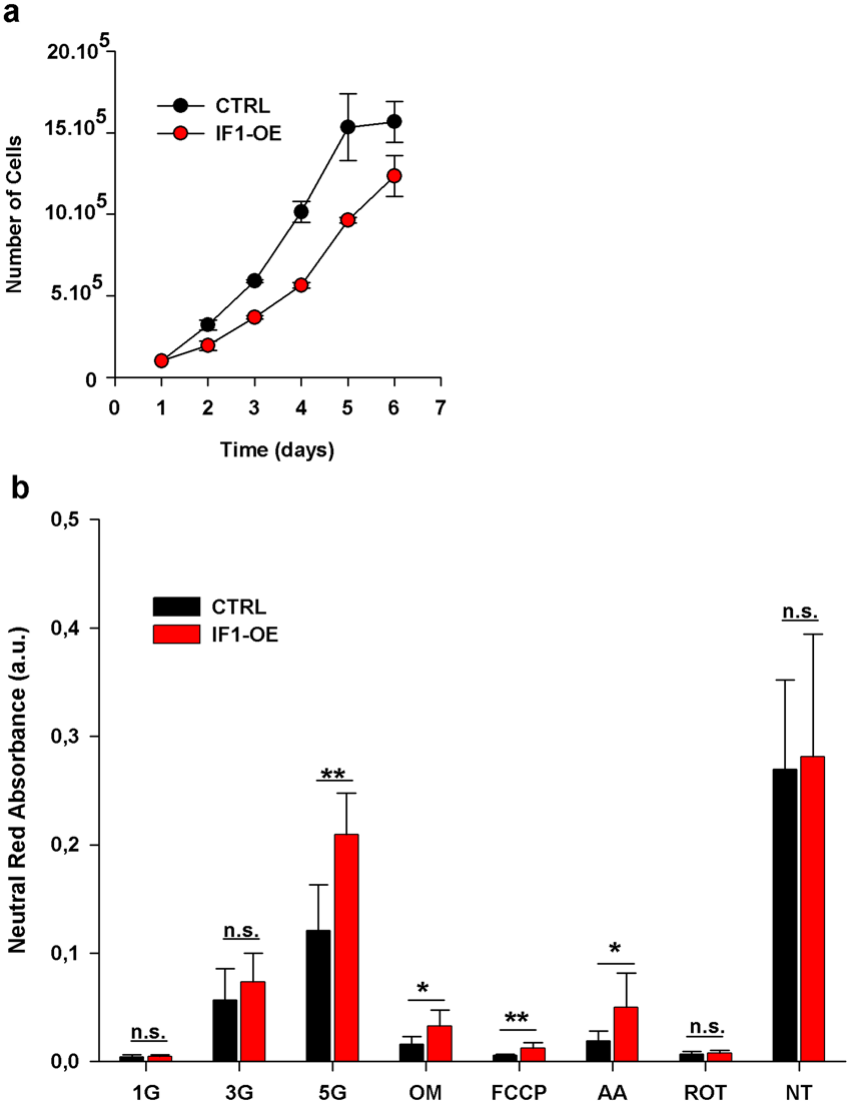
(**a**) Growth curve of INS-1E overexpressing IF1 (IF1-OE) and control INS-1E cells (CTRL). (**b**) Neutral red cell viability assay in INS-1E overexpressing IF1 (IF1-OE) and control INS-1E cells (CTRL). Cells were incubated at low glucose concentrations 1 mmol/l (1 G), 3 mmol/l (3 G), and 5 mmol/l (5 G) overnight (16 h) in the modified culturing medium. When indicated, cells were treated with 5 µM rotenone (ROT), 5 µM antimycin A (AA), 5 µM FCCP, 5 µM oligomycin (OM) for 2 hours. INS-1E cells incubated at standard 11 mM glucose were used as non-treated (NT) controls. Student’s t-test was applied to compare the statistical difference between the control and IF1-overexpressing group. p values are set as follows: *p < 0.05, **p < 0.005, ***p < 0.0005.

To observe the effect of IF1 on cellular metabolism in model pancreatic β-cells (INS-1E), a stable cell line overexpressing native IF1 was generated. 2.5 fold increase in IF1 protein compared to controls was estimated by western blot (see Supplementary Fig. S1). We first characterized this cell line regarding its growth rate and viability. IF1-overexpressing cells showed a 33% decrease in cell growth rate compared to controls (assessed between the 1^st^ and 3^rd^ day) (Fig. 1a). Viability of IF1-overexpressing cells was not significantly affected under normal conditions when cultured in standard medium with 11 mM glucose (Fig. 1b). Two-hour incubation in the presence antimycin A (inhibitor of Complex III), oligomycin (inhibitor of ATP synthase), or uncoupler of the respiratory chain, FCCP (carbonyl cyanide-p-trifluoromethoxyphenylhydrazone), reduced the viability in both groups nonetheless IF1-overexpressing cells remained more viable than the corresponding controls. Incubation with Complex I inhibitor, rotenone, substantially reduced the viability of both studied group and no difference between them was observable. Lowering glucose to 5 mM for 16 hours caused a decline in viability in both groups, but again IF1 overexpressing cells were more resistant. A more dramatic decrease in glucose concentration (3 and 1 mM) led to a further decline in cell viability in both groups and gradually eliminated the differences between IF1 overexpressing and control cells. Thus apparently, under the majority of stressful situations, IF1-overexpressing cells are less vulnerable and better adapted to functional damage of respiratory chain or reduced ATP levels. Similar effects were previously described in neurons and hepatocytes overexpressing IF1, where induction of signalling cascades preventing cell death was reported^28,30^.

### IF1 overexpression downregulates cellular respiration in INS-1E cells

**Figure 2:**
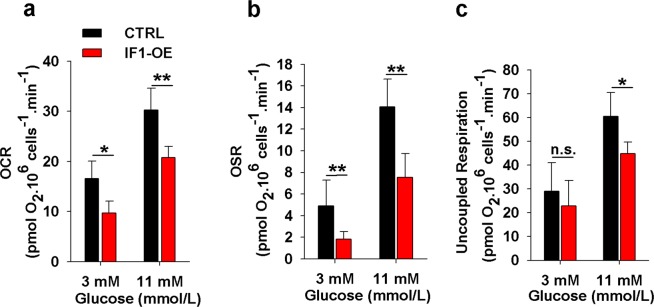
Cellular respiration of IF1-overexpressing and control INS-1E cells preincubated for 2 hours in a KRH buffer with indicated glucose concentrations: (**a**) Basal respiration, (**b**) Oligomycin-sensitive respiration, (**c**) Uncoupled respiration. Student’s t-test was applied to compare the statistical difference between the control and IF1-overexpressing group. p values are indicated as follows: *p < 0.05, **p < 0.005, ***p < 0.0005.

IF1-overexpressing cells displayed low oxygen consumption rates (OCR), accounting for 68% of rate for control cells in media with 11 mM glucose and 59% in media with 3 mM glucose (Fig. 2a). The overexpression of native IF1, therefore, caused the opposite effect than previously reported for IF1 silencing in INS-1E cells^33^. The ATP synthase inhibitor oligomycin further decreased these rates even in IF1-overexpressing cells, demonstrating that IF1 overexpression was not sufficient to fully inhibit ATP synthesis (Fig. 2b). The maximum respiration capacity was examined by the successive additions of the artificial uncoupler carbonyl cyanide-p-trifluoromethoxyphenylhydrazone (FCCP). Interestingly, these maximum rates of respiration were also slightly decreased in IF1-overexpressing cells (Fig. 2c). This might be explained by previously reported tight coupling of ATP synthesis and the rate of oxidative metabolism in INS-1E^34^. It seems that inhibition/stimulation of ATP synthesis also adjusts the rate of upstream catabolic reactions. In support, we observed that the rate of NADH increase, after inhibition of the respiratory chain, is lower in IF1-overexpressing cells compared to controls (see Supplementary Fig. S2).

### IF1 overexpression downregulates cellular ATP levels

**Figure 3:**
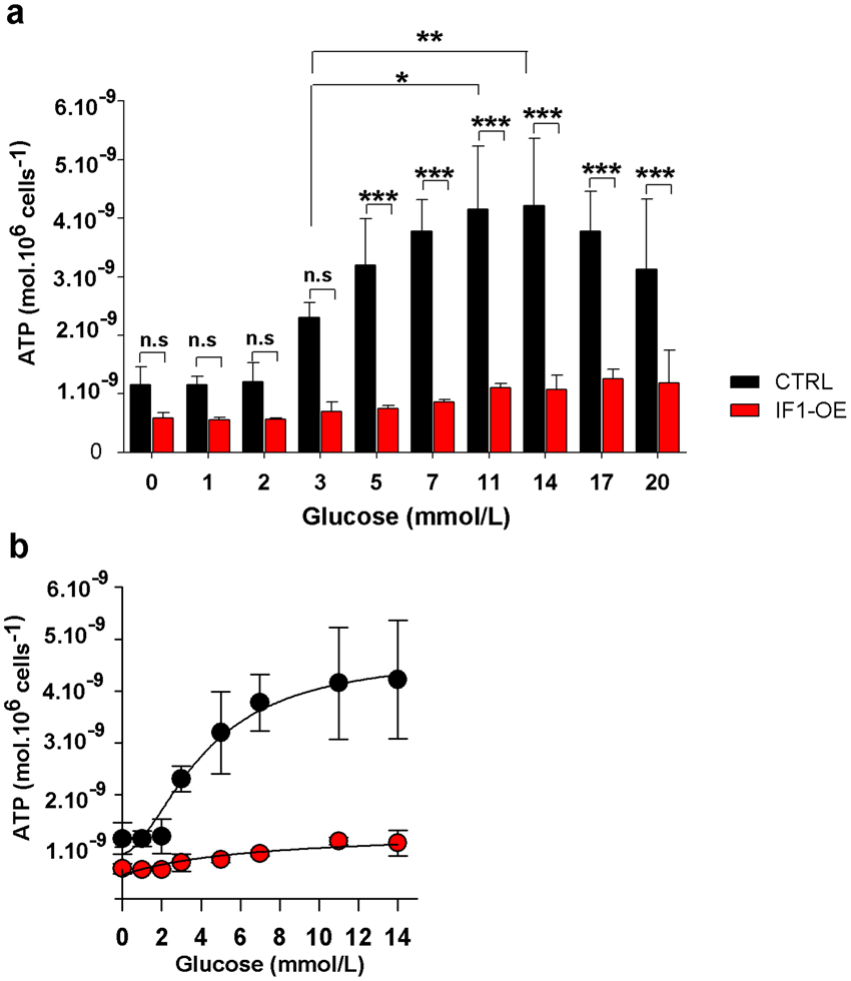
(**a)** Cellular ATP levels in IF1-overexpressing and control INS-1E cells preincubated for 2 hours in a KRH buffer with indicated glucose concentrations. (**b)** Glucose/ATP dose-response curve was generated by fitting the data (in the range between 0 and 14 mM glucose) to a 4-parameters logistic curve. Student’s t-test was applied to compare the statistical difference between control and IF1-overexpressing group. p values are indicated as follows: *p < 0.05, **p < 0.005, ***p < 0.0005.

To estimate total cellular ATP in cell lysates, we used a luciferase assay. ATP levels were dramatically decreased in IF1-overexpressing cells, especially at glucose levels higher than 2 mM glucose (Fig. 3a). As expected, in control cells, ATP levels were gradually elevated with increasing glucose concentration. This increase was significantly abrogated by the IF1 overexpression. When plotted using four-parameters logistic curves, the glucose dose-response curve for control cells had a typical sigmoidal shape with the maximum ATP production rate 3.5·10^−9^ mol·10^6^ cells^−1^ and EC50 was estimated as 4.1 mM. In IF1-overexpressing cells, V_max_ was 8.5·10^−9^ mol·10^6^ cells^−1^, and EC50 was 6.4 mM (Fig. 3b). These results clearly show that the native IF1 protein, produced by overexpression, is active and capable of downregulating ATP production by the ATP synthase.

### IF1 overexpression downregulates *in situ* free cytosolic and mitochondrial ATP levels

**Figure 4:**
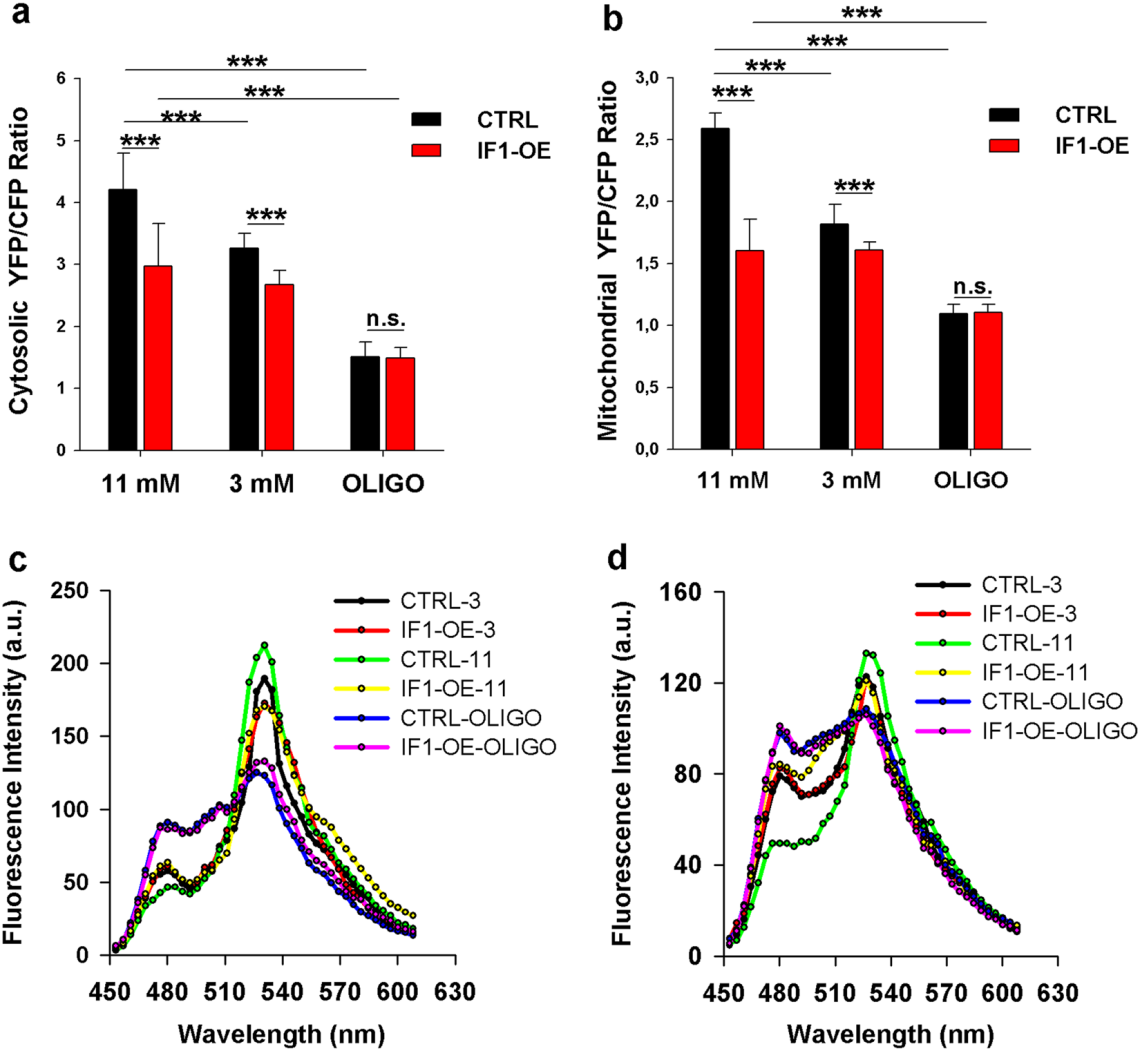
Confocal microscopy of ATeam biosensor was used to determine levels of free unbound ATP in cytosol (**a**) and mitochondria (**b**) of INS-1E cells preincubated for 2 hours at 3 or 11 mM glucose. 5 µM Oligomicin was added when indicated. A minimum of 10 cells was analysed from each group. ANOVA followed by posthoc Tukey’s multiple comparisons tests were applied. p values are set as follows: *p < 0.05, **p < 0.005, ***p < 0.0005. Representative fluorescence spectra of ATeam biosensor localized to cytosol **(c)** and mitochondria **(d)**.

In pancreatic β-cells, a substantial amount of ATP is expected to be compartmentalized within the insulin granules, thus not actively participating in cellular metabolism^35^. To validate differences in unbound ATP concentrations in IF1-overexpressing cells, we employed the FRET-based biosensor ATeam^36^. IF1-overexpressing cells and corresponding controls were transfected with ATeam targeted either to cytosol or mitochondria of these cells. Emission spectra of ATeam were monitored using confocal microscopy (Fig. 4c,d), and the ratio between the maximum CFP and YFP fluorescence provided an estimation of free unbound ATP levels. Both in cytosol and mitochondria, the concentration of free ATP was diminished in IF1-overexpressing cells at 11 mM glucose. At 3 mM glucose, the differences were lower but still significant (Fig. 4a,b). To validate the ability of the probe to monitor ATP concentrations, INS-1E cells were treated with an inhibitor of ATP synthase, oligomycin, for 1 h. This treatment led to a further decrease in observed free ATP levels, as anticipated. Moreover, the free ATP levels were equal in control and IF1-overexpressing cells after oligomycin treatment. Representative images of cytosolic and mitochondrial ATeam are shown in the Supplement (Figs. S3, S4).

### IF1 overexpression does not change mitochondrial morphology and cristae ultrastructure in INS-1E cells

**Figure 5:**
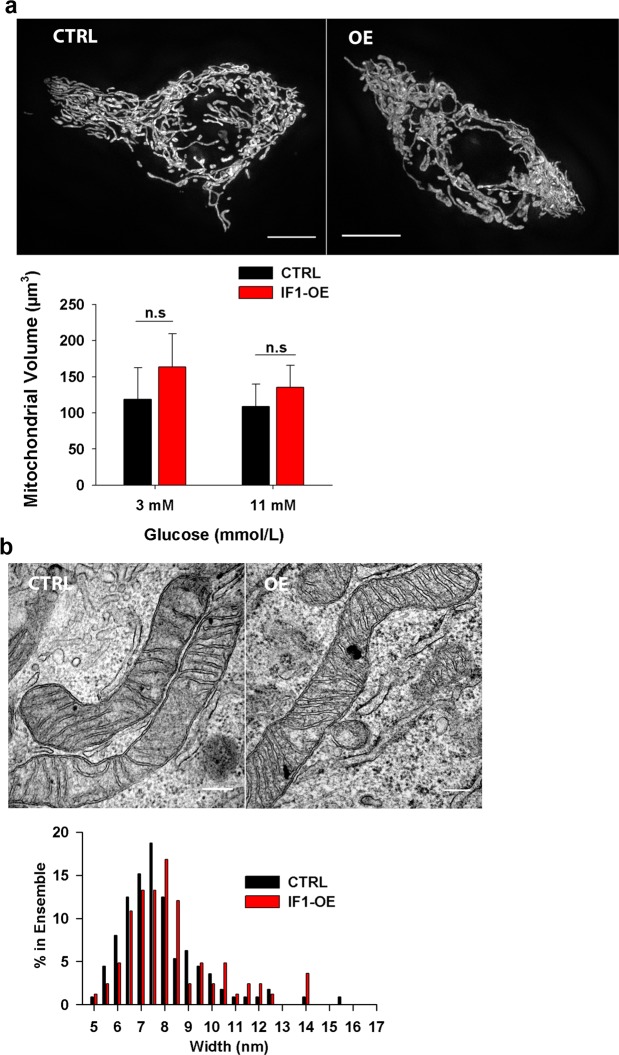
(**a**) Visualisation of mitochondrial network morphology (labelled with roGFP addressed to mitochondria) in IF1-overexpressing and control INS-1E cells by SIM microscopy. Scale bars 5 µm. Chart bar represents the quantitative analysis of mitochondrial volume by Amira 5.4.5 software. Student t-test was applied to compare the statistical difference between control and IF1-overexpressing group. p values are indicated as follows: *p < 0.05, **p < 0.005, ***p < 0.0005. (**b)** TEM microscopy of mitochondria in IF1-overexpressing and control INS-1E cells. Scale bars: 200 nm. Mitochondrial cristae width was analysed by FIJI software and shown as a histogram.

Mitochondrial morphology is tightly connected to the β-cell function^37^. We, therefore, next evaluated the effect of IF1 overexpression on mitochondrial network volume and morphology (Fig. 5a). IF1-overexpressing cells contained mainly well‐connected tubular mitochondria similarly as control cells (see Supplementary Fig. S5 for mitochondrial length analysis). Amira analysis of fluorescence SIM images revealed that the average volume and width of mitochondrial tubule was not significantly changed. Likewise, TEM studies showed a similar arrangement of cristae in IF1-overexpressing cells and no differences were observed in the distribution of cristae widths (Fig. 5b).

### IF1 overexpression diminishes insulin secretion

**Figure 6:**
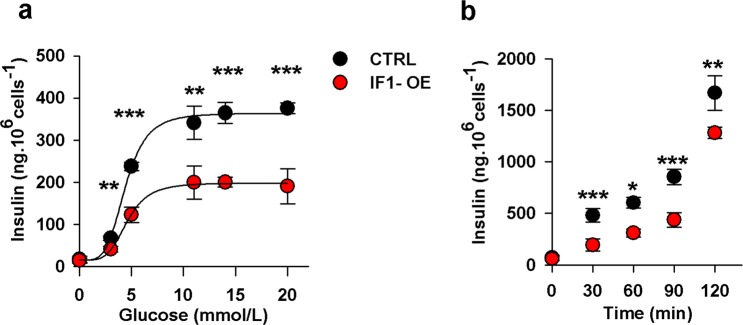
(**a**) Insulin secretion by IF1-overexpressing and control INS-1E cells pre-incubated for 1 hour in a KRH buffer with indicated glucose concentrations. Data were fitted to a 4-parameters logistic curve. (**b)** Time-dependence of insulin secretion in cells incubated with standard 11 mM glucose. Student’s t-test was applied to compare the statistical difference between control and IF1-overexpressing group. p values are indicated as follows: *p < 0.05, **p < 0.005, ***p < 0.0005.

Our previous study demonstrated that downregulation of IF1 by siRNA enhances insulin secretion, especially at low glucose levels. To observe whether overexpression of native IF1 has the opposite effect, we pre-incubated IF1-overexpressing cells with increasing glucose levels for 1 hour and measured the amount of insulin secreted into the media. The obtained data revealed that GSIS was profoundly reduced in cells overexpressing IF1 (Fig. 6a). We plotted the insulin secretion in the range from 5 to 15 mM glucose by the four-parameter logistic curve to obtain the dose-response relation. By that, we estimated that EC50 was 4.4 mM and the maximum insulin release rate was 364 ng.10^6^ cells^−1^ in control cells. In IF1-overexpressing cells, EC50 was 4.6 mM, and the plateau of saturated insulin release rate decreased to 198 ng.10^6^ cells^−1^.

To examine time-dependent changes in insulin secretion, we measured insulin secretion at 11 mM glucose at several time points (Fig. 6b). We observed that at all studied time points (apart from the t = 0 s) insulin secretion was significantly reduced in IF1-overexpressing cells (p < 0.05).

### PKA activation abolishes inhibitory effect of IF1

**Figure 7:**
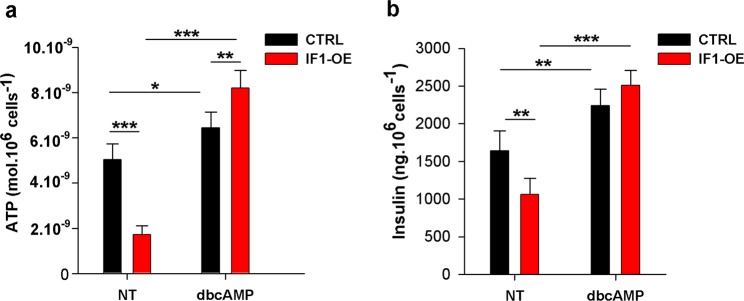
Cellular ATP levels **(a)** and insulin secretion **(b)** in IF1-overexpressing and control INS-1E cells treated with 200 µM dibutyryl cAMP (dbcAMP) for 8 hours. As non-treated controls, we used INS-1E cells incubated at standard medium with 11 mM glucose. ANOVA followed by posthoc Tukey’s multiple comparisons tests were applied. p values are indicated as follows: *p < 0.05, **p < 0.005, ***p < 0.0005.

Phosphorylation of IF1 at serine 39 by protein kinase A (PKA) prevents its binding to the ATP synthase^38^. Therefore, we investigated whether the activation of PKA will reverse the metabolic changes induced by IF1 overexpression. IF1-overexpressing INS-1E cells and corresponding controls were incubated with dibutyryl cAMP (dbcAMP) for 8 hours, and total cellular ATP levels and insulin secretion were evaluated. We found that dbcAMP upregulated ATP levels 4.7-fold in IF1-overexpressing cells and only 1.3-fold in controls, both groups reaching similar ATP levels in the end (Fig. 7a). Similarly, insulin levels increased 2.4 fold in IF1-overexpressing cells and only 1.4 fold in control cells (Fig. 7b) approaching similar levels in both cell types.

To sum up, activation of the PKA pathway augments the ATP production and GSIS in INS-1E. Due to the much higher effect of dbcAMP treatment in IF1-overexpressing cells, we conclude that PKA activation is able to abolish the inhibitory effect of IF1 protein. This might be mediated directly due to the previously reported ability of PKA to phosphorylate IF1 on Ser 39, or some other indirect mechanism might be involved.

### IF1 overexpression increases the amount and size of insulin granules

**Figure 8:**
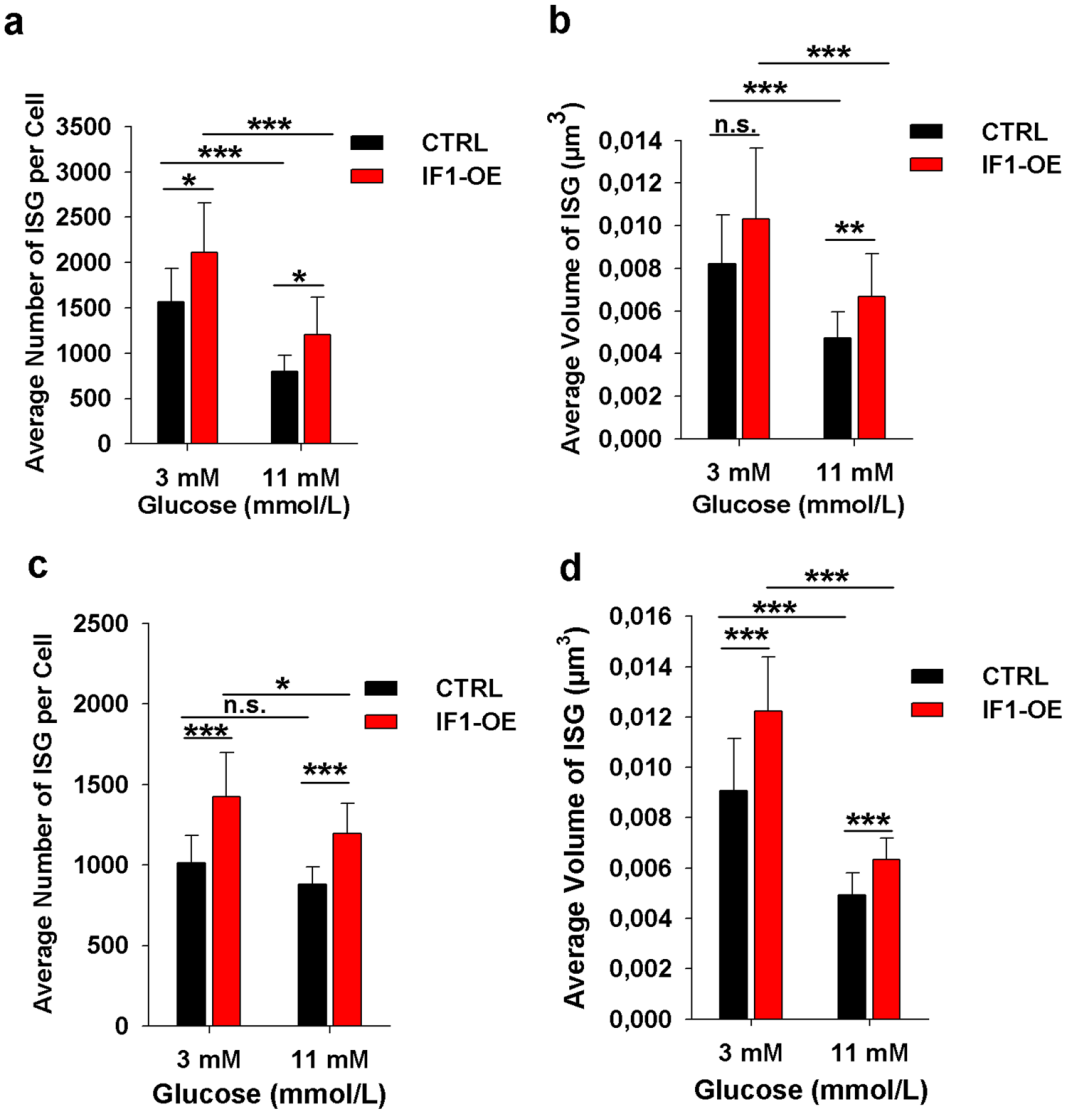
Quantitative analysis of immuno-fluorescently labelled insulin granules visualized by SIM microscopy. Amira 5.4.5 software was used to determine the amount **(a,c)** and the average volume of insulin granules **(b,d)**. Cells were incubated at indicated glucose concentrations in KRH buffer for either 2 hours **(a,b)** or 30 minutes **(c,d)**. A minimum of 18 cells was analysed from each group. ANOVA followed by posthoc Tukey’s multiple comparisons tests were applied. p values are indicated as follows: *p < 0.05, **p < 0.005, ***p < 0.0005.

We next used immunocytochemistry with anti-insulin antibody to estimate the total amount of insulin secretory granules present in control and IF1-overexpressing INS-1E cells. Since the granules are often positioned in close distances and their diameter is often below 300 nm, a high-resolution microscopy technique has to be employed to estimate their amount in the cells. We used structured illumination microscopy (SIM) followed by Amira software analysis of insulin granule amount and size (see Supplementary Fig. S6 for representative images). Cells were incubated with low (3 mM) glucose and standard (11 mM) glucose for 2 hours prior to fixation. We found that control INS-1E cells contained on average 797 insulin granules and incubation with low (3 mM) glucose caused a 97% increase in their amount (Fig. 8a). IF1 overexpression augmented the number of granules above the levels in control cells, leading to a 34% increase in the number of insulin granules at standard glucose and 35% at low glucose conditions.

Increases in the number of granules were accompanied by an increase in their size. Their average volume in the control cells was 4.7·10^−3^ µm^3^ while in IF1-overexpressing cells, the volume increased to 6.7·10^−3^ µm^3^ (Fig. 8b). After incubation with low glucose, the average volume was 8.2·10^−3^ µm^3^ in control cells and 10.3·10^−3^ µm^3^ in IF1-overexpressing cells. Individual histograms of insulin granule volumes distributions can be found in the Supplement (Figs. S7, S8).

Interestingly, also shorter (30 min) incubation periods induced the above-described changes (Fig. 8c,d) though the difference in insulin granules number between low and high glucose concentrations was less evident.

Overall, our data demonstrate that suppressed secretion of insulin due to low glucose or IF1 overexpression leads to adaptive response inducing greater content of retained insulin in the INS-1E cells.

### Downregulation of ATP levels and insulin secretion by transient transfection of IF1

**Figure 9:**
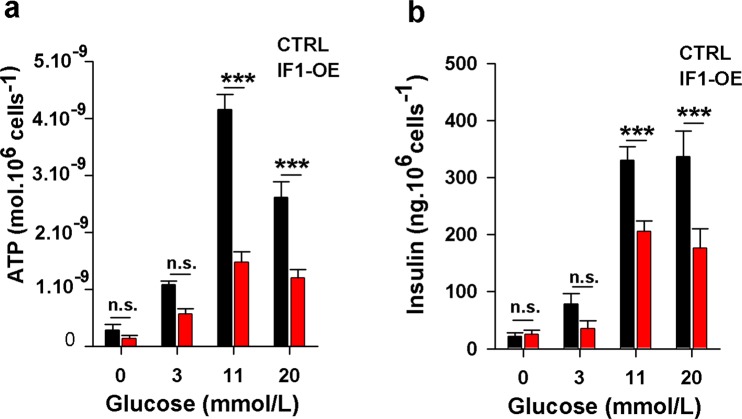
INS-1E cells transiently transfected with IF1-expressing plasmid and empty pCMV6 (control cells). Cells were preincubated for 2 hours in a KRH buffer with indicated glucose concentrations and subsequently, total cellular ATP levels were determined **(a)** or insulin secretion was followed **(b)**. ANOVA followed by posthoc Tukey’s multiple comparisons tests were applied. p values are indicated as follows: *p < 0.05, **p < 0.005, ***p < 0.0005.

To further confirm the results obtained with IF1-overexpressing stable cell line, we also examined the impact of transient transfection of IF1 in INS-1E cells. INS-1E cells were transfected with native-IF1 expressing plasmid and control cells were transfected with empty pCMV6 plasmid. Two days after transfection cells were incubated at several glucose concentrations for 2 hours and ATP and insulin secretion was determined. In agreement with previous results, transient overexpression of IF1 caused a profound decrease in ATP levels by 66% and 54% at 11 and 20 mM glucose, respectively (Fig. 9a). Consistently, insulin secretion was reduced by 38% at 11 mM glucose and by 47% at 20 mM glucose. At low glucose (3 mM) these differences were still observable but to a lesser extent (Fig. 9b). We conclude that transient overexpression of native IF1 can significantly reduce ATP production and insulin secretion in INS-1E and has a similar effect as stable overexpression of IF1.

### Regulation of endogenous IF1 levels by glucose concentration

**Figure 10:**
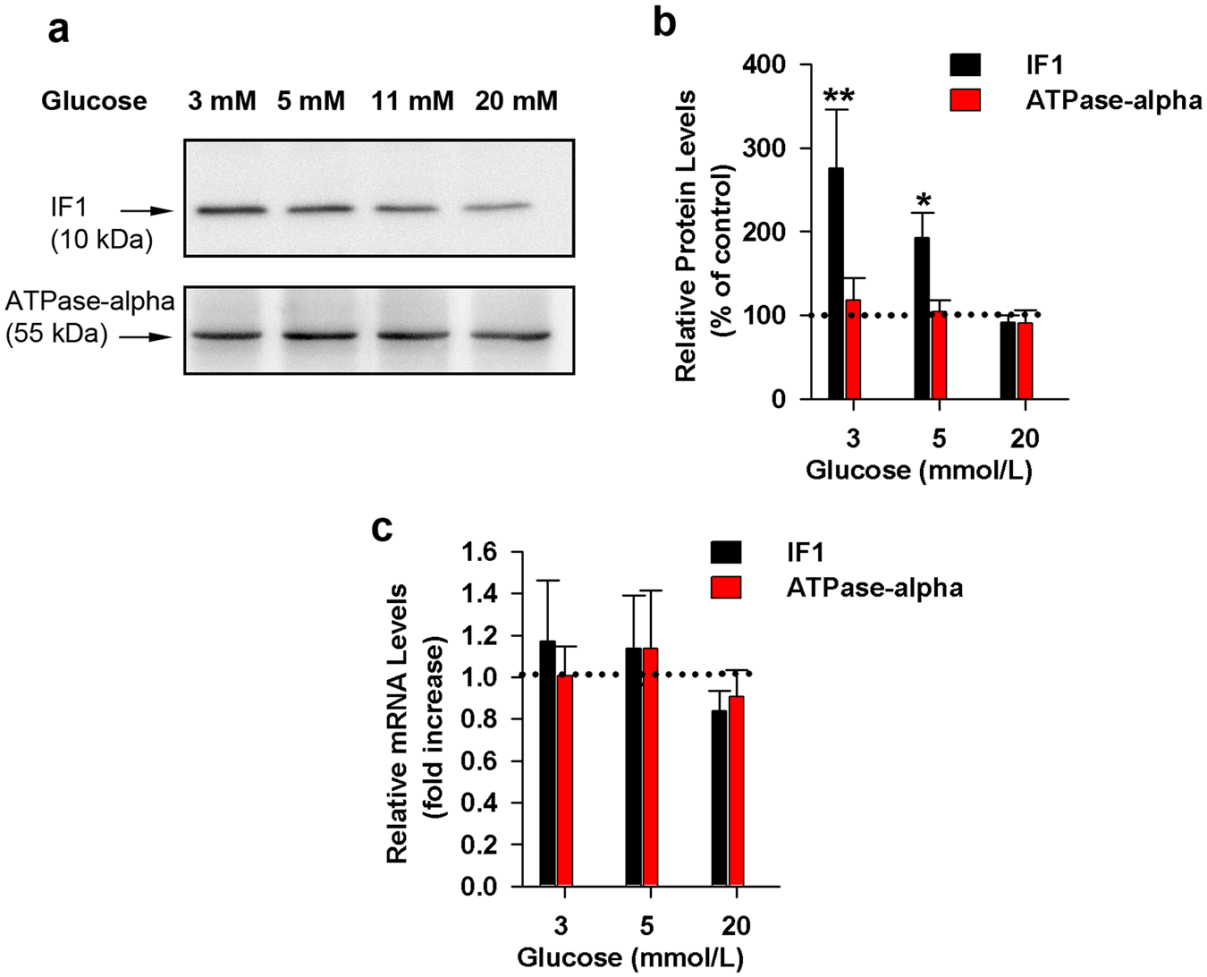
Effect of glucose concentrations on relative IF1 protein levels in INS-1E cells. Cells were incubated for 8 h with modified culturing medium with adjusted glucose levels. As non-treated controls, we used INS-1E cells incubated at standard 11 mM glucose. (**a)** Representative western-blot, (**b)** quantification of protein levels. (**c)** Relative mRNA levels in INS-1E cells incubated for 8 h with modified culturing medium with adjusted glucose levels. As non-treated controls, we used INS-1E cells incubated at standard 11 mM glucose. Significance was determined by the Kruskal*–*Wallis’s test followed by posthoc Dunnett’s multiple comparisons to control. p values are indicated as follows: *p < 0.05, **p < 0.005, ***p < 0.0005.

Since we demonstrated that modulation of IF1 proteins levels has the potential to adjust insulin secretion, we studied changes of endogenous IF1 protein levels in response to the varying glucose concentrations. We found that incubation of INS-1E cells with low glucose (3 mM and 5 mM) for 8 hours in modified culture media induces upregulation of IF1 protein levels compared to cells maintained in 11 mM or 20 mM glucose (Figs. 10a,b; S9). Since the α-subunit of ATP synthase did not change, also the IF1 to ATP synthase α-subunit stoichiometry increased at low glucose. We further investigated whether similar changes can be observed in IF1 mRNA levels by quantitative RT-PCR (qRT-PCR). We were not able to detect any significant changes in IF1 mRNA levels based on the glucose availability in the incubation media (Fig. 10c). This finding is consistent with previous studies reporting that IF1 protein levels are adjusted mainly on post-transcriptional level^39^. Hypothetically, specific IF1 degradation might also be retarded at low glucose.

We conclude that INS-1E cells regulate the amount of IF1 protein in mitochondria based on glucose availability in order to adjust ATP production and consequently, insulin secretion.

## Discussion

The role of IF1 in cancer metabolism is attracting attention since the IF1 overexpression was reported in several frequently occurring human carcinomas like lung or colon. However, the regulation and the functional role of IF1 in pancreatic β-cells are entirely understudied, although ATP synthesis and its regulation are critical for GSIS. Our work is the first to demonstrate that overexpression of native IF1 reduces insulin secretion in model pancreatic β-cells INS-1E by decreasing ATP levels and overall cellular metabolism. We show that native IF1 overexpressed in INS-1E cells is active and capable of downregulating ATP levels. The effect of oligomycin, an artificial inhibitor of ATP synthesis, was nevertheless still observable, thus indicating that ATP synthesis was not entirely inhibited by IF1-overexpression. The prominent effect of IF1 on ATP levels in INS-1E cells can be attributed to the fact that ATP in INS-1E is nearly completely derived from the oxidative phosphorylation^40^. The present data strongly support the possibility that IF1 can also inhibit ATP synthesis besides the well-established inhibition of ATP hydrolysis. In agreement, the recently published study by Gu *et al*.^32^ reported that the tetramers of ATP synthase internally linked by the dimers of IF1 (between the two F1 sectors of neighbour dimers) appear to be in the inhibited state as judged based on the presence of tightly bound MgADP to all the β_DP_ and β_TP_ subunits.

Several previous studies followed the effect of IF1 overexpression in primary mouse cells or cancer cell lines. Of note, in the majority of these studies, the mutated form of the pH-insensitive IF1 (H49K mutant) was employed^41^. *In vitro* studies show that the H49K mutant of IF1 assembles into an active dimer at neutral pH, while the native IF1 is thought to be inactive tetramer at pH around neutrality^42–48^. However, it is plausible that the cellular environment might shift the pH of these oligomeric transitions.

In INS-1E cells, a substantial amount of ATP is supposed to be enclosed within the insulin granules^35^. This ATP does not have a regulatory function in cellular processes. It is therefore important to study also levels of free unbound ATP. We employed the FRET-based biosensor ATeam to show that also free ATP levels are suppressed by the IF1 overexpression.

As expected, a profound decrease in ATP levels led to suppressed insulin secretion in IF1-overexpressing cells in the range of 5 to 20 mM glucose. We, therefore, confirmed that IF1 is a negative regulator of GSIS as proposed in our previous study^33^.

Upregulation of protein kinase A (PKA) activity is considered as a promising target in the therapy of type 2 diabetes mellitus (T2DM) due to its pleiotropic effects on glycemic control^7^. We observed that PKA activation by dibutyryl cAMP treatment completely abolished the inhibitory effect of IF1 on ATP synthesis and insulin secretion. We conclude that IF1 inhibitory activity can be prevented by PKA stimulation and consequently that IF1 might function as one of the key effectors of PKA signalling in pancreatic β-cells.

As confirmed by the recent work resolving the structure of ATP synthase tetramer, IF1 is involved in ATP synthase oligomerization. In turn, ATP synthase dimerization and oligomerization are established to determine mitochondrial cristae morphology, since the ATP synthase dimers form the cristae rims^49,50^. This is enabled by the ability of ATP synthase dimers to bend the lipid bilayer and thus form the sharp cristae rims. Consistently, the role of IF1 in mitochondrial ultrastructure and cristae density in mitochondrial tubule was previously reported in HELA cells^51^. In our study, the impact of IF1 overexpression on mitochondrial ultrastructure was not detectable by TEM and also the gross morphology of the mitochondrial network, examined by SIM microscopy, did not differ. Possibly, a thorough 3D electron microscopy examination would be needed to reveal more subtle changes.

However, super-resolution studies of insulin granules revealed an evident increase in insulin granules number and volume in IF1-overexpressing cells. This might represent an adaptive response enhancing the available pool at simultaneously reduced GSIS. Similar changes were induced by incubation of cells at low (3 mM) glucose, again indicating that the reduced secretion of insulin leads to its higher accumulation within pancreatic β-cell.

Remarkably, we also found out that IF1 protein levels in INS-1E are adjusted based on the glucose concentration in the incubation media, which further supports the regulatory role of IF1 in GSIS. Under low glucose levels, IF1 protein is endogenously upregulated.

To sum up, one can predict that misregulated IF1 expression may affect insulin secretion in pancreatic β-cells. Too low levels of IF1 can lead to excessive GSIS even at low non-stimulatory glucose levels. In contrast, upregulated IF1 levels and maybe also downregulated PKA signalling would suppress or delay insulin secretion. All these events would thus contribute to T2DM pathogenesis.

In conclusion, it appears that the physiological role of IF1 in pancreatic β-cells is critical for appropriate insulin secretion and should be of great interest. Further studies using animal models or human pancreatic islets are thus absolutely essential.

## Methods

### Chemicals

All chemicals were purchased from Sigma Aldrich unless otherwise stated.

### Cell culture

Rat insulinoma INS-1E cells were cultured as described previously^52^. Briefly, cells were grown with 11 mM glucose in RPMI 1640 medium supplemented also with 1 mM pyruvate, 2 mM L-glutamine, 10 mM HEPES, 5% (v/v) fetal calf serum, 50 μM β-mercaptoethanol, 50 IU/ml penicillin, and 50 μg/ml streptomycin. All cell lines were maintained at 37 °C, 5% CO_2_ in a humidified incubator. When indicated cells were preincubated before experiment in KRH buffer (135 mM NaCl, 3.6 mM KCl, 10 mM HEPES, 0.5 mM MgCl_2_, 1.5 mM CaCl_2_, 0.5 mM NaH_2_PO_4_, pH 7.4) containing 0.1% fatty acid free (FAF) BSA. For long-term incubations (8 h or longer) with adjusted glucose levels modified culturing medium was used (standard culturing medium without glucose, pyruvate, glutamine with fetal calf serum reduced to 2.5%).

### INS-1E cells overexpressing native IF1

INS-1E cells were transfected with Origene plasmid (RN207924) by use of Lipofectamine 3000 reagent according to the manufacturer’s instructions. Cells stably overexpressing native rat IF1 were selected by geneticin at concentration 50 µg/ml. Overexpression of IF1 was confirmed by SDS PAGE followed by western blot. Simultaneously the stable cell line expressing the empty pCMV6-Entry vector (Origene, PS100001) was generated as a control cell line.

### Cell growth

Cell growth was determined after seeding 1·10^5^ cells in RPMI media and culturing the cells for up to 144 h. At selected time points, cells were trypsinized, stained with trypan blue and viable cells were counted.

### Neutral red assay

Cells were seeded at a 96-well plate, and after 4 days, the assay was performed. Cells were incubated at low glucose concentrations 1 mmol/l (1 G), 3 mmol/l (3 G), and 5 mmol/l (5 G) overnight (16 h) in the modified culturing medium. Cells were treated with 5 µM rotenone (ROT), 5 µM antimycin A (AA), 5 µM FCCP, 5 µM oligomycin (OM) for 2 hours. After incubation with neutral dye, cells were washed with PBS to remove the unabsorbed dye and fixed by CaCl_2_ with formaldehyde. Dye absorbed by cells was released using 1% acetic acid with ethanol. The intensity of released dye was measured spectrophotometrically at 540 nm. The wavelength of 630 nm served for background measurements.

### SDS PAGE and western blot

Cells were lysed in 50 mM HEPES, 135 mM NaCl, 1 mM EDTA, 1% Triton X-100, and 1 mM PMSF (pH 7.4). Amount of protein was quantified by bicinchoninic acid assay (BCA). 20 µg of proteins were separated on SDS-polyacrylamide gels. Separated proteins were then transferred by a wet electro blot onto PVDF membranes. Membranes were incubated with desired primary antibody (IF1 (ab110277, Abcam), ATP synthase α-subunit (ab 14748, Abcam)) followed by the secondary (horseradish peroxidase-conjugated) antibody. Before ECL detection, the membrane was incubated with Luminata Forte Western HRP Substrate (Millipore Corporation, Billerica, MA 01821). ECL light intensity was quantified by densitometry with FIJI^53^.

### Cellular ATP levels

Total cellular ATP was determined with ATP Assay bioluminescence kit HS II (Roche Diagnostics GmbH, Manheim, Germany). Samples were prepared according to the manufactures instructions. Thus cells were lysed by boiling in EDTA buffer (100 mM Tris, 4 mM EDTA, pH 7.75) for 2 minutes. Bioluminescence was determined with Luminometer Synergy HT (Bio-TEK) microplate reader.

### Oxygen consumption

Oxygen consumption was measured with high-resolution respirometer Oxygraph-2k (Oroboros, Innsbruck, Austria) in KRH buffer. Cells were preincubated for 2 hours in KRH buffer (135 mM NaCl, 3.6 mM KCl, 10 mM HEPES, 0.5 mM MgCl_2_, 1.5 mM CaCl_2_, 0.5 mM NaH_2_PO_4_, pH 7.4) containing 0.1% fatty acid free (FAF) BSA. The desired final glucose concentration was adjusted before each measurement. Oligomycin sensitive respiration (OSR) was determined after addition of 1 µM oligomycin. At the end of each run, respiratory activity was titrated by trifluoromethoxy carbonylcyanide phenylhydrazone (FCCP) and the maximum respiratory chain capacity has been derived from the maximum rate achieved in a titration.

### *In situ* monitoring of cytosolic and mitochondrial ATP

INS-1E cells overexpressing IF1 were transfected with ATeam plasmid or ATeam plasmid targeted to the mitochondrial matrix^35^ by use of lipofectamine 2000 as described in the manufacturer’s protocol. ATeam fluorescence was monitored with confocal microscope Leica SP5 in lambda scan mode (XYλ). Excitation was set to 405 nm, and emission was collected between 453–608 nm with detection bandwidth 5 nm and lambda detection step size 3.88 nm. Intensity corresponding to maxima was determined and used for further calculations. When indicated 10 µM oligomycin was added and cells incubated for 1 h.

### Insulin release

Cells were seeded at 0.3·10^6^ cells/well in poly-l-lysine-coated 12-well plates 2 days before the experiment. Cells were washed with KRH buffer without glucose and then incubated in KRH with 0.1% FAF BSA with desired glucose concentrations. After 1 h, insulin levels in the media were determined using the Rat Insulin High Sensitivity ELISA kit (BioVendor, Brno, Czech Republic).

### Structured Illumination Microscopy (SIM)

To quantify the number of insulin granules, we fixed the cells in 4% paraformaldehyde for 15 min, washed them twice in PBS, and incubated them for 15 min with 5% donkey serum. Cells were incubated with anti-insulin mouse monoclonal antibody (ab6995, Abcam, 1:300 dilution) for 1 h at room temperature with constant shaking. Coverslips were then washed 3 times in PBS and incubated for 1 h in PBS with the Alexa-Fluor-488 secondary antibody (Thermo Scientific – Life Technologies) followed by 3 washes in PBS. Samples were mounted in the glycerol buffer (refractive index 1.42) containing antifade reagent. To study mitochondrial morphology, INS-1E cells were transfected with GFP targeted to the mitochondrial matrix, and the next day fixed and mounted as described above.

High-resolution fluorescence microscopy was carried out using a DeltaVision OMX™ with the Blaze SIM Module (GE Healthcare) equipped with a 60 × 1.42, PlanApo N, Oil Immersion objective, 405 nm, 488 nm and 568 nm laser lines, and the OMX Standard filter set drawer. Images were acquired in structured illumination mode using a Z-spacing of 125 nm and reconstructed using Softworx software (GE Healthcare, Seattle, WA, USA).

### Transmission electron microscopy

For transmission electron microscopy (TEM), cells were fixed for 2 hours in 2.5% glutaraldehyde and 2% paraformaldehyde in 0.1 M cacodylate buffer (pH 7.2), washed and postfixed in 2% OsO_4_ in the same buffer. Fixed cells were dehydrated through an ascending ethanol series and embedded in Araldite - Poly/Bed^®^ 812 mixture. Thin sections were cut on a Reichert-Jung Ultracut E ultramicrotome and stained using uranyl acetate and lead citrate. Sections were examined and photographed using a JEOL JEM-1011 electron microscope. Then, the cristae lumen width was quantified with FIJI^53^.

### qPCR

Primers were designed using NCBI Design Primer tool - for subunit α of ATP synthase: 5′–TCC AAG CAG GCT GTT GCT TAC–3′ (forward); 5′–TGT AGG CGG ACA CAT CACCA–3′ (reverse), for β actin: 5′–GAT CAA GAT CAT TGC TCC TCCTG–3′ (forward); 5′–ACG CAG CTC AGT AAC AGTCC–3′ (reverse), for IF1: 5′–AAG GCT GAA GAG GAT CGGTA–3′ (forward); 5′–TTC ATG GTG CTT CTT CAA GGCA–3′ (reverse). The qPCR reaction was performed in LightCycler 480 (Roche) utilizing Forget-Me-Not™ EvaGreen^®^ qPCR Master Mix (Biotium, CA, USA). The absolute mRNA amounts were calculated from crossing points of each run.

### Statistical analysis

Data are presented as means ± S. D. of four to six biological replicates. Statistical analysis was performed by running the SigmaPlot 9.0 (Systat Software, Chicago, IL, USA) or GraphPad Prism 8. (GraphPad, La Jolla, CA, USA). Specific tests are described at figure legends. Statistical significance was defined as follows: *p < 0.05, **p < 0.005, ***p < 0.0005.

## Supplementary information

Supplementary Information

## Data Availability

The datasets generated during and/or analysed during the current study are available from the corresponding author on reasonable request.
