# Supplementary material for: Overexpression of native IF1 downregulates glucose-stimulated insulin secretion by pancreatic INS-1E cells
Source: Sci Rep. 2020 Jan 31;10:1551. doi: 10.1038/s41598-020-58411-x (PMC6994519; doi:10.1038/s41598-020-58411-x)
Supplement: Supplementary file 1 — Supplementary Information [file 41598_2020_58411_MOESM1_ESM.pdf]

Supplemental Information

Scientific Reports

Overexpression of native IF1 downregulates glucose-stimulated insulin secretion by pancreatic INS-1E cells

Anežka Kahancová, Filip Sklenář, Petr Ježek and Andrea Dlasková

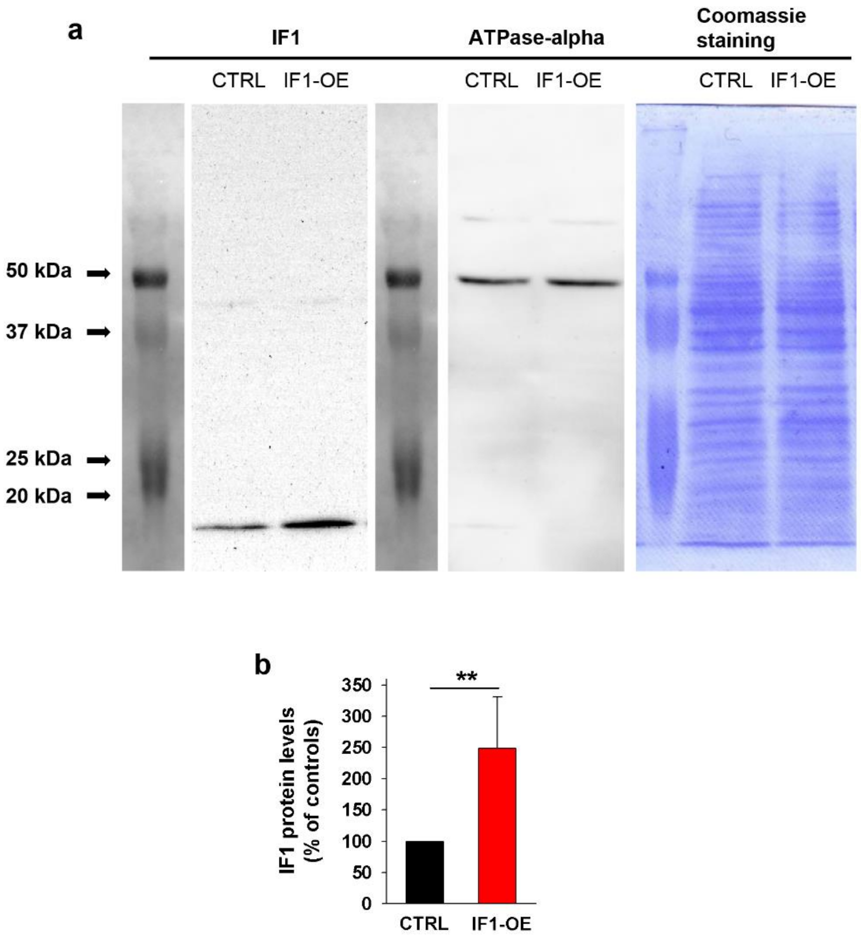

Supplementary fig. S1 Western blots of IF1 protein and ATP synthase α-subunit in IF1-overexpressing (IF1-OE) INS-1E cells. The membrane was stained by Coomassie blue **(a)**. Quantification of relative amounts of each protein was done in FIJI **(b)**.

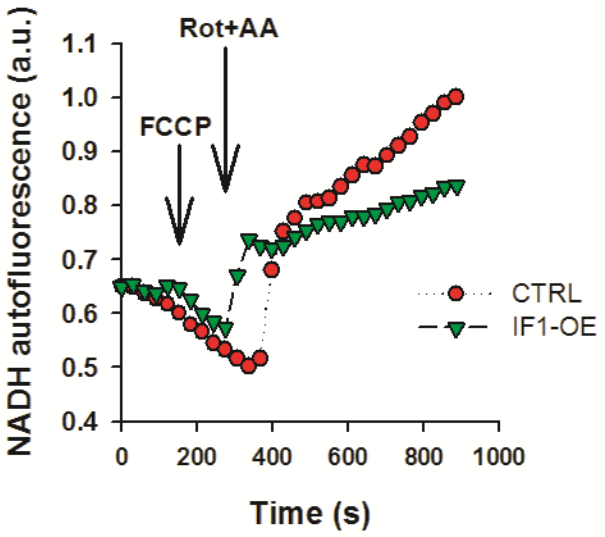

Supplementary fig. S2 NADH autofluorescence was monitored by 2-photon confocal microscopy. Excitation was set at 740 nm, and emission was collected at 467–499 nm. When indicated, an uncoupler of the respiratory chain, 5 μM FCCP, was added. Afterwards, inhibitors of the respiratory chain, 5 μM rotenone together with 10 μM antimycin A, was added.

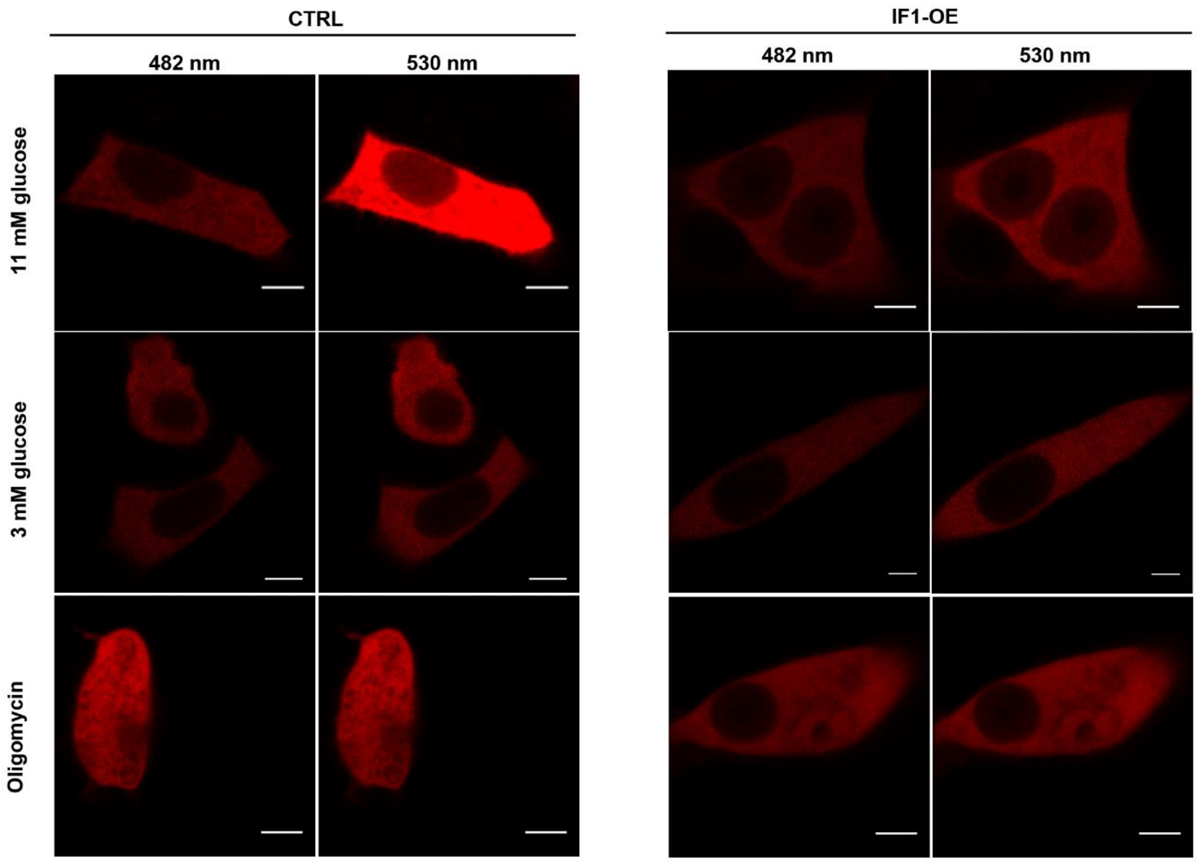

Supplementary fig. S3 Representative images of cytosolic ATeam fluorescence at 482 nm and 530 nm. Cells were incubated for 2 hours in KRH buffer with 3 or 11 mM glucose. When indicated, 5 μM oligomycin was added. Scale bars represent 5μm.

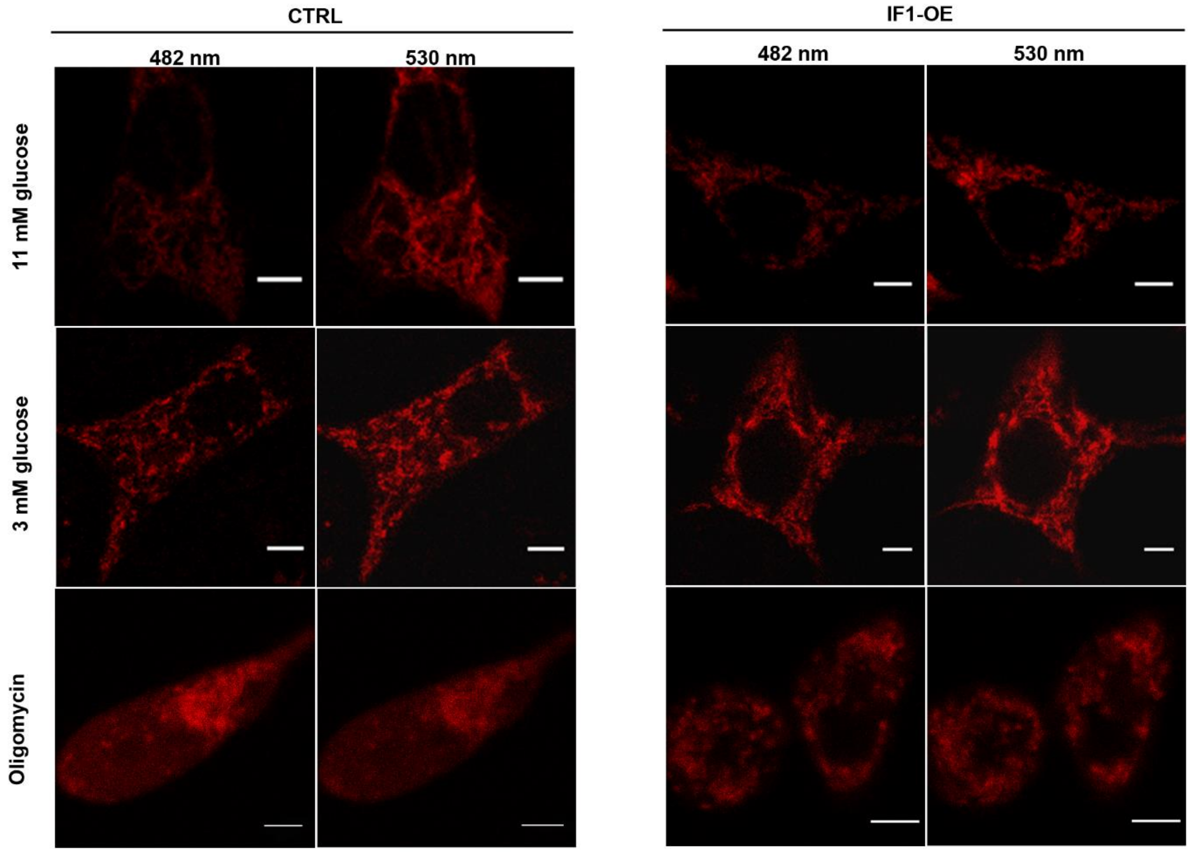

Supplementary fig. S4 Representative images of mitochondrial ATeam fluorescence at 482 nm and 530 nm. Cells were incubated for 2 hours in KRH buffer with 3 or 11 mM glucose. When indicated, 5 μM oligomycin was added. Scale bars represent 5μm.

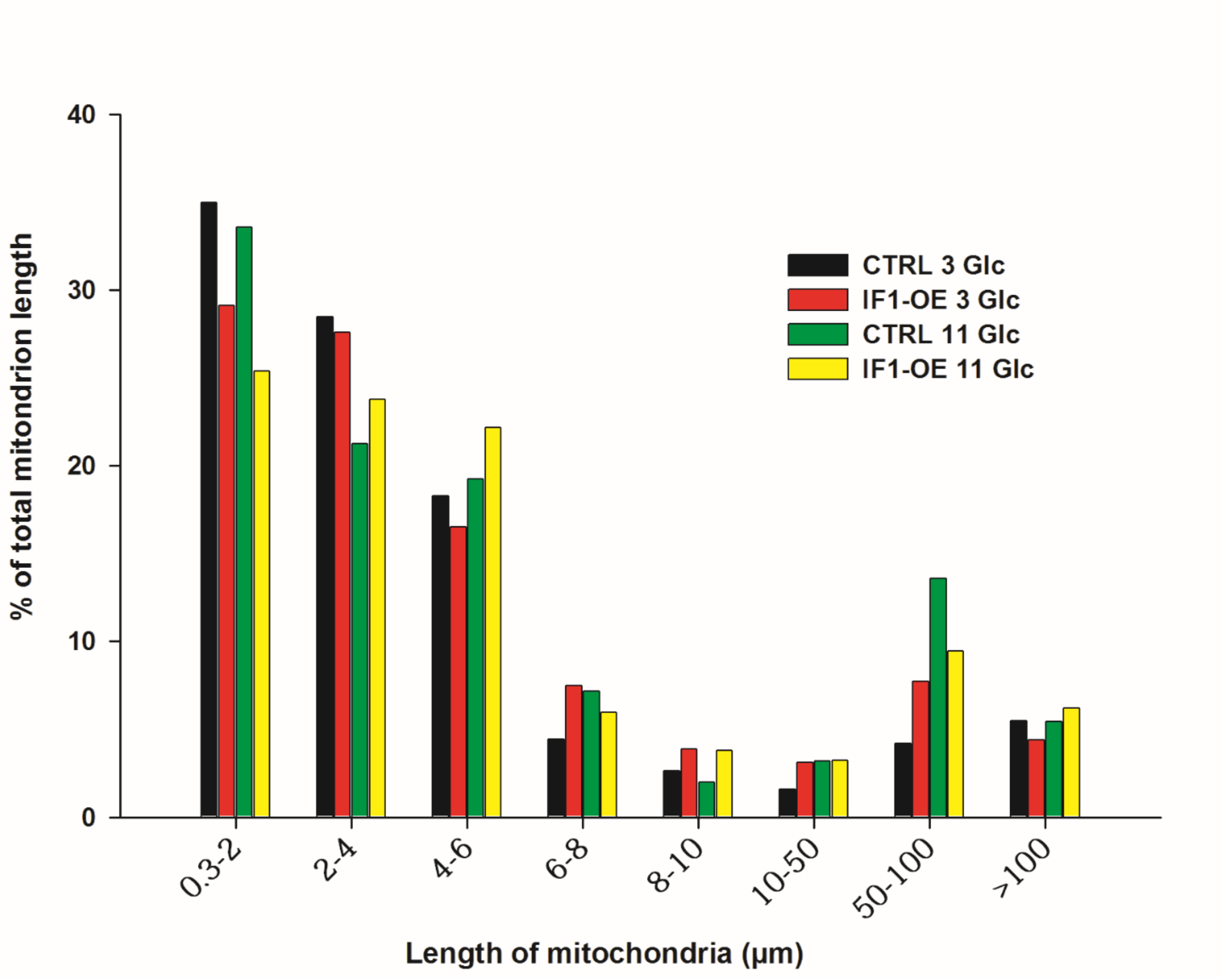

Supplementary fig. S5 Amira 5.4.5 analysis of mitochondrial network length in IF1-overexpressing and control INS-1E cells. IF1-overexpressing cells and control cells were transfected with redox GFP targeted to mitochondria. 2 days after transfection cells were preincubated for 2 hours in KRH buffer with either 3 mM or 11 mM glucose, then fixed and the mitochondrial network was visualised by SIM microscopy.

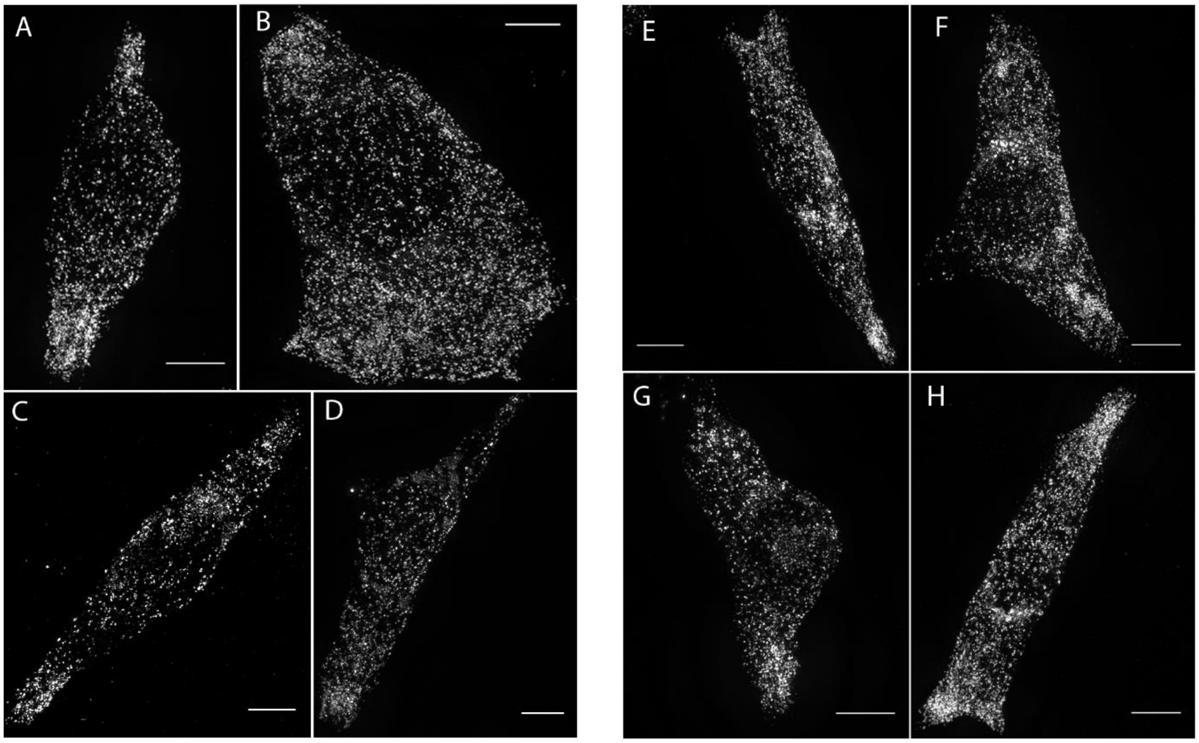

Supplementary fig. S6 Representative images of immuno-labelled insulin secretory granules (ISG) in IF1-overexpressing and control INS-1E cells as visualized by SIM microscopy. Scale bars: 5 µm. **A)** CTRL cells preincubated for 2 h in 3 mM glucose, **B)** IF1-overexpressing cells preincubated for 2 h in 3mM glucose, **C)** CTRL cells preincubated for 2 h in 11mM glucose, **D)** IF1-overexpressing cells preincubated for 2 h in 11mM glucose. CTRL cells preincubated for 2 h in 3 mM glucose, **E)** CTRL cells preincubated for 30 min in 3 mM glucose, **F)** IF1-overexpressing cells preincubated for 30 min in 3mM glucose, **G)** CTRL cells preincubated for 30 min in 11mM glucose, **H)** IF1-overexpressing cells preincubated for 30 min in 11mM glucose.

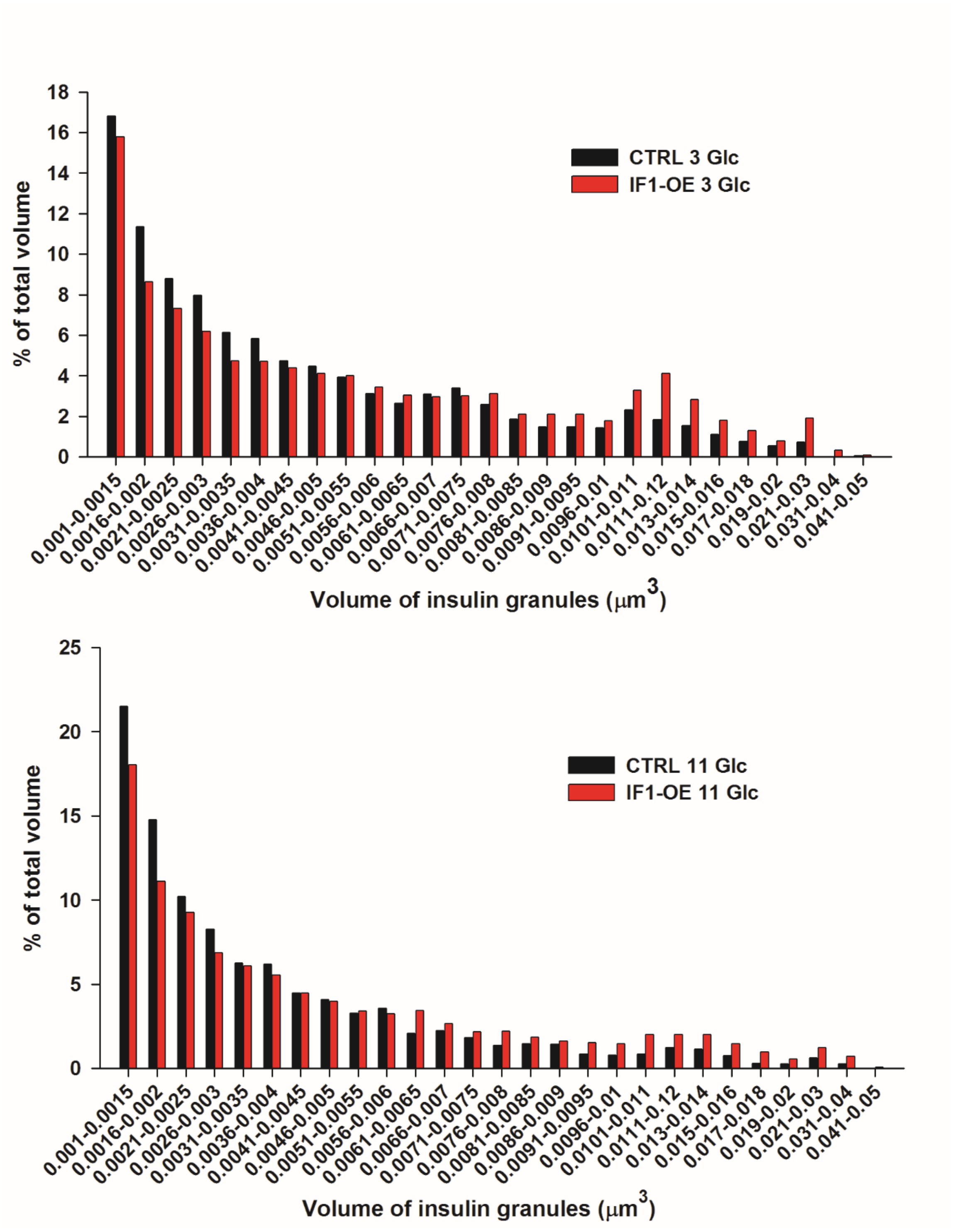

Supplementary fig. S7 Histograms of insulin granules volumes determined by AMIRA 5.4.5 software from SIM images of ISG (see figure S6). Before imaging, cells were preincubated for 2 hours in KRH buffer with 3 or 11 mM glucose.

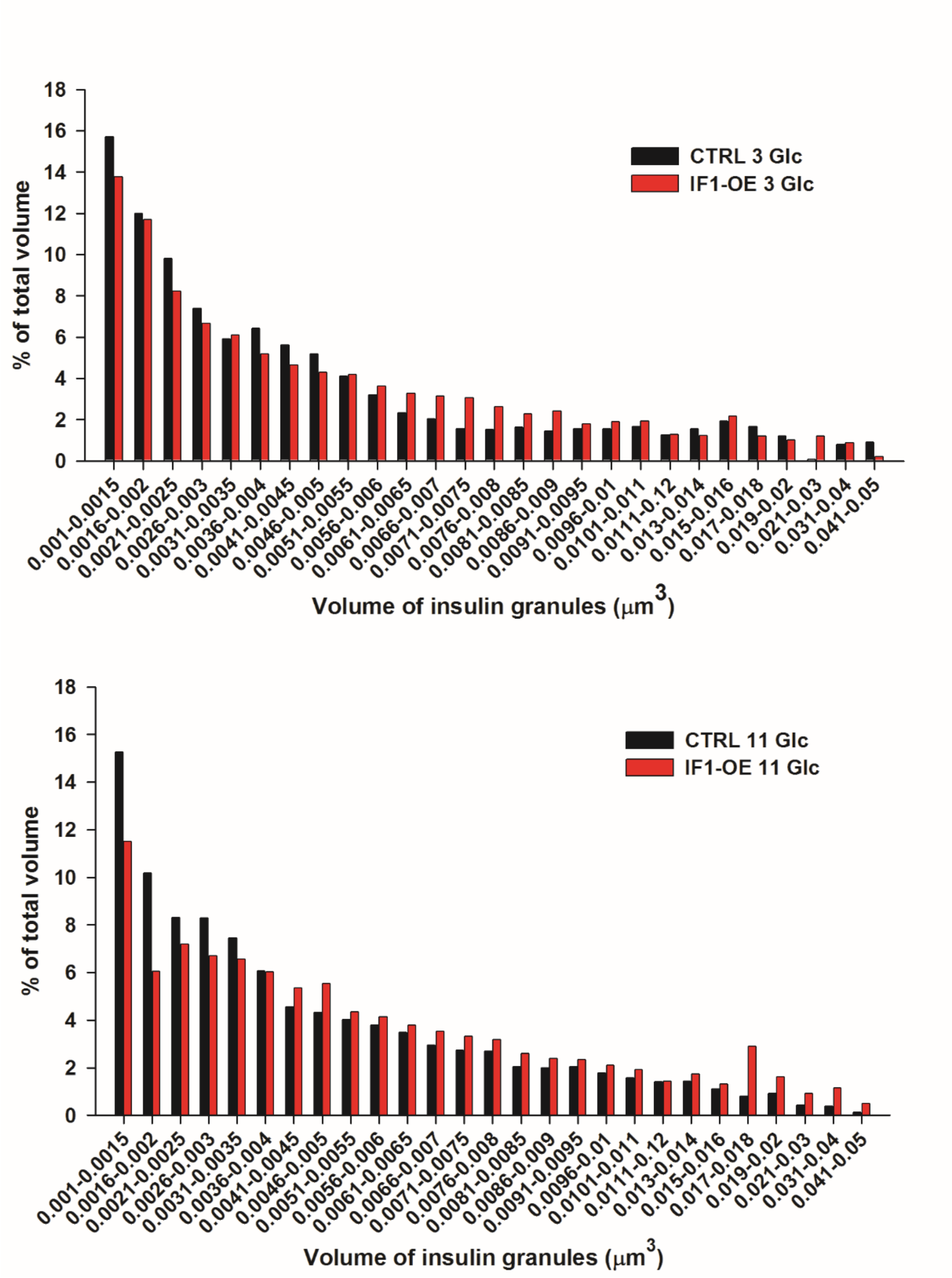

Supplementary fig. S8 Histograms of insulin granules volumes determined by AMIRA 5.4.5 software from SIM images of ISG (see figure S6). Cells were preincubated for 30 minutes in KRH buffer with 3 or 11 mM glucose before imaging.

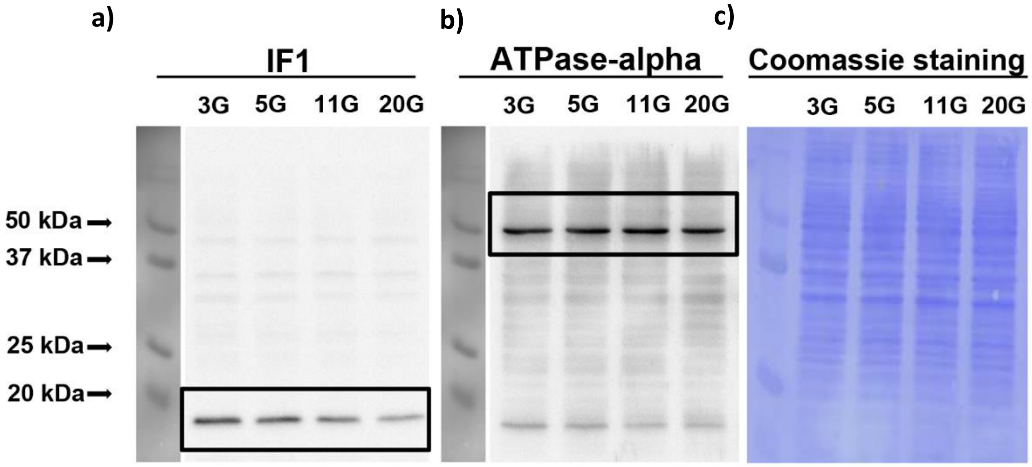

Supplementary fig. S9 Full-length western blots of IF1 protein **(a)** and ATP synthase alpha subunit **(b)** in INS-1E cells incubated for 8 h with modified culturing medium with adjusted glucose levels. The membrane was stained by Coomassie blue **(c).** Cells were incubated at defined glucose concentrations 3 mmol/l (3G), 5 mmol/l (5G), 11 mmol/l (11G) and 20 mmol/l (20G) overnight (8 h) in the modified culturing medium.
